# Supplementary material for: Learning the diffusion of nanoparticles in liquid phase TEM via physics-informed generative AI
Source: Nat Commun. 2025 Jul 8;16:6298. doi: 10.1038/s41467-025-61632-1 (PMC12238262; doi:10.1038/s41467-025-61632-1)
Supplement: Supplementary file 1 — Supplementary Information [file 41467_2025_61632_MOESM1_ESM.pdf]

Supplementary Information
Learning the Diffusion of Nanoparticles in Liquid Phase TEM via Physics-informed Generative AI

Zain Shabeeb[1], Naisargi Goyal[1], Pagnaa Attah Nantogmah[1], Vida Jamali[1*]

[1]School of Chemical and Biomolecular Engineering, Georgia Institute of Technology, 311 Ferst Drive, Atlanta, 30332, Georgia, USA.

*Corresponding author. E-mail: vida@gatech.edu;

Contents

1 Experimental Sample Preparation **S2**
1.1 Chemicals and materials S2
1.2 Gold nanorod solutions S2

2 Training Datasets **S2**

3 LEONARDO model **S3**
3.1 Model architecture S3
3.2 Model training S6
3.3 Model validation S6

4 Supporting Tables **S7**

5 Supporting Figures **S8**

1 Experimental Sample Preparation

1.1 Chemicals and materials

Hexadecyltrimethylammonium bromide (CTAB, $\geq$ 98%), Gold (III) Chloride Trihydrate ($HAuCl_4$, $\geq$ 99.9%), Silver Nitrate ($AgNO_3$, $\geq$ 99.0%), Sodium Borohydride ($NaBH_4$, 99%) and L-Ascorbic Acid (99%) were purchased from Sigma-Aldrich. Hydrochloric acid (HCl, 36.5-38.0%) and Nitric acid (HNO_3, 70%) were purchased from Fischer Scientific. Chemicals were wrapped in Aluminum foil and stored in a vacuum desiccator at room temperature. 18.2 Ω-cm deionized water (DI water, Millipore) was used for preparing all aqueous solutions. All chemicals were of reagent grade and used without further purification.

1.2 Gold nanorod solutions

Gold nanorods (AuNRs) were synthesized following a modified seed-mediated growth protocol [1]. Prior to synthesis, all glassware and stir bars were washed with aqua regia (3:1 HCl: HNO_3 by volume), followed by rinsing with copious amounts of DI water. For the synthesis, first, the seed solution was prepared by pipetting 250 μL of $HAuCl_4$ (10 mM), followed by 600 μL ice-cold $NaBH_4$ (10 mM), into an aqua-regia washed glass vial containing 7 mL of CTAB (100 mM) under vigorous stirring. A color change from colorless to golden orange was observed upon the addition of $HAuCl_4$, and then to golden brown upon the addition of $NaBH_4$. The seed solution was stirred for 2 minutes and then allowed to age undisturbed for an hour. Next, the growth solution was prepared by adding the following materials in the listed order to an aqua-regia washed conical flask containing 100 mL CTAB (100 mM) under vigorous stirring: 4.25 µL $HAuCl_4$ (10 mM), 650 μL $AgNO_3$ (10 mM), 675 μL Ascorbic Acid (100 mM), 4 mL seed solution. A color change from golden orange to colorless was observed upon the addition of Ascorbic acid, and a gradual color change to dark purple was observed upon the addition of the seed solution. The resulting solution was allowed to age for 12 hours to complete the growth process.

The AuNRs were purified by centrifuging the growth solution at 15365 $\times g$ for 25 minutes and discarding the supernatant. The sedimented AuNRs were redispersed with DI water 5$\times$ the volume of the AuNRs and centrifuged a second time at 20913 $\times g$ for 20 minutes. The supernatant was discarded, and the sedimented AuNRs were wrapped in foil and refrigerated for long-term storage. The synthesized rods were polydisperse in length, suitable for covering a range of particle sizes in this study.

To complement this sample with a larger rod size of $L \geq 50$, we also synthesized larger AuNRs following a seed-mediated growth protocol published in the literature [2]. The seed solution was prepared by mixing 10 mL of 0.1 M CTAB solution with 100 μL of 25 mM $HAuCl_4$ in a 20 mL vial and under vigorous stirring. Next, 600 μL of 10 mM ice-cold NaBH4 was rapidly pipetted into the Au-CTAB solution and stirred for 2 minutes. The seed solution was then left undisturbed at room temperature for 30 min prior to use in the next step. The growth solution was prepared by mixing 3.6 g of CTAB and 0.4936 g of NaOL in 196 mL of DI water in a 500-mL aqua-regia-washed conical flask. The solution was then heated with agitation until all the CTAB was dissolved. The mixture was allowed to cool down to 30 °C, at which point 1.45 mL of 10 mM $AgNO_3$ was added under stirring at 700 rpm for 15 minutes. Next, 4 mL of 25 mM $HAuCl_4$ was added to the mixture and kept undisturbed at room temperature for 1.5 hours. After that, 840 μL of HCl was added to the solution and the mixture was stirred at 400 rpm for 15 minutes. Lastly, 500 μL of 0.064 M Ascorbic acid was injected into the growth solution, and the mixture was vigorously stirred at 1,200 rpm for 30 seconds. 80 μL of the seed solution was then injected, and the solution was stirred for 30 seconds before being left undisturbed at room temperature for 12 hours to complete the growth process. 40 mL of the final products were isolated by centrifugation at 6829 $\times g$ for 15 minutes, followed by careful removal of the supernatant. The sedimented rods were redispersed in 50 mL of DI water for long-term storage. For the preparation of the liquid phase transmission electron microscopy (LPTEM) sample, 1 mL of the stock solution was diluted by adding 5 mL of DI water before loading it onto the chip.

The 40 nm gold nanorods used in the particle size study in Section 2.4 of the main paper were obtained from NanoComposix, characterized by a peak wavelength of 660 nm and were supplied with a citrate ligand.

2 Training Datasets

The trajectories of particle motion obtained from processing the in situ videos were a time series of the x and y coordinates of the particles in each frame. To formulate the training dataset, these trajectories of various lengths were segmented into shorter 200-frame-long trajectories. To augment the training set with trajectories that reflect motion at longer time scales, the originally processed trajectories were also

sub-sampled at rates from 2 to 60, and then segmented. For example, a sub-sampling rate of 2 means that every second x and y coordinate in the trajectory was extracted to form a new trajectory, which was then segmented into 200-time-point trajectories. The x and y components were treated as a combined 2D trajectory. A total of $38,279$ trajectories from LPTEM videos were collected for training.

The 200-frame experimental trajectories were normalized to lie between 0 and 1 for model training. The normalization process was performed as follows: each trajectory was first centered by subtracting the minimum value of all time frames for the x and y axes. The centered trajectory was then normalized by dividing by the range of values across the x and y axes. Mathematically, the normalization can be expressed as:

$$\mathbf{r}_{\text{normalized}} = \frac{\mathbf{r} - \min_t \mathbf{r}}{\max_{t,i} \mathbf{r} - \min_{t,i} \mathbf{r}} \tag{S1}$$

where the subscript t refers to the time frame, the subscript i refers to the axes, $\min_t \mathbf{r}$ denotes the per-axis minima, $\min_t \mathbf{r} = \left[\min_t x_t \;\; \min_t y_t\right]$, and $\max_{i,t} \mathbf{r}$ and $\min_{i,t} \mathbf{r}$ denote the maximum and minimum values across all entries in $\mathbf{r}$.

3 LEONARDO model

3.1 Model architecture

Figure S3 shows the architecture of LEONARDO, which is a variational autoencoder with the encoder and decoder adapted from the Transformer architecture [3] that maps an input trajectory of 200 time frames, $\mathbf{r} = (r_1, r_2, \cdots, r_{T=200})$, where $\mathbf{r} = (x, y)$ represents the position vector of the nanoparticle with x and y denoting the x and y coordinates of the particle's position, respectively, to a sequence of continuous representation $\mathbf{z} = (z_1, \cdots, z_{12})$. Given $\mathbf{z}$, the decoder generates an output trajectory $\hat{\mathbf{r}} = (\hat{r}_1, \cdots, \hat{r}_{200})$. Here is the detail of each block:

First, a batch of N input trajectories is passed through a convolutional layer L to increase the embedding dimensions of each trajectory from 1 to 128 and get to $\mathbf{X}$ that is a tensor of size $N \times 128 \times 200$, where N is the batch size. $\mathbf{X}$ is the input to the encoder block depicted in Figure S3.

Encoder. The encoder consists of two parts. First, the tensor $\mathbf{X}$ goes through a multi-headed self-attention layer (detailed by Vaswani et al. [3]) with 8 heads to capture the time dependencies within a trajectory, followed by layer normalization and fully connected feed-forward layers. The attention layer maps a query, $\mathbf{Q}$, and a set of key-value pairs, $\mathbf{K}, \mathbf{V}$, to an output. The multi-head attention layer allows the model to jointly attend to information from different representation subspaces at different positions along the length of the trajectory.

$$\begin{aligned} &\text{Multihead}(\mathbf{Q}, \mathbf{V}, \mathbf{K}) = \text{concatanate}(\text{head}_1, \cdots, \text{head}_8)\mathbf{W}^{\mathbf{O}}, \\ &\text{where } \; \text{head}_i = \text{Attention}(\mathbf{Q}\mathbf{W}^{\mathbf{Q}}{}_i, \mathbf{K}\mathbf{W}^{\mathbf{K}}{}_i, \mathbf{V}\mathbf{W}^{\mathbf{V}}{}_i), \end{aligned} \tag{S2}$$

where $\mathbf{W}^{\mathbf{O}} \in \mathbb{R}^{dh_v \times d_{\text{model}}}, \mathbf{W}^{\mathbf{Q}} \in \mathbb{R}^{d_{\text{model}} \times d_k}, \mathbf{W}^{\mathbf{K}} \in \mathbb{R}^{d_{\text{model}} \times d_k}$, and $\mathbf{W}^{\mathbf{V}} \in \mathbb{R}^{d_{\text{model}} \times d_v}$ are parameter matrices of the model. The attention matrix used here is the standard scaled Dot-Product attention [3] that is computed as:

$$\text{Attention}(Q, K, V) = \text{softmax}(\frac{QK^T}{\sqrt{d_k}})V. \tag{S3}$$

Here, we employed $h = 8$ attention heads; therefore, $d_v = d_k = d_{\text{model}}/8 = 32$. The fully connected feed-forward layers deployed in the attention layer of the encoder block consist of two linear, fully connected transformations with a ReLU activation in between:

$$\text{FFN}(\mathbf{x}) = \max(0, \mathbf{x}\mathbf{W}_1 + \mathbf{b}_1)\mathbf{W}_2 + \mathbf{b}_2, \tag{S4}$$

where $\mathbf{x}$ is the input, $\mathbf{W}_1$, and $\mathbf{W}_2$ are weight matrices, and $\mathbf{b}_1$ and $\mathbf{b}_2$ are bias vectors. The output of this transformer block goes through another identical transformer block before entering the second part of the encoder block, which is a series of convolutional layers that reduce the size of the tensor to the latent space dimension. The first convolutional layer has a kernel size of 7, a stride of 1, and a padding of 3 to reduce the embedding dimension from 128 to 32. The second convolutional layer has a kernel size of 2 and a stride of 2 to reduce the size of the last tensor dimension from 200 to 100. This tensor is flattened to a size of 3200 before being further reduced in a linear layer to a size of 512.

The next few operations are adapted from the standard variational autoencoder [4]. In this stage, the encoder generates two vectors of size 12, $\boldsymbol{\mu}$ and $\log(\boldsymbol{\sigma}^2)$, representing the mean and log-variance of the latent space distribution. These vectors are used to sample the latent variable $\mathbf{z}$ via the reparameterization trick:

$$\mathbf{z} = \boldsymbol{\mu} + \boldsymbol{\sigma} \cdot \boldsymbol{\epsilon}, \tag{S5}$$

where $\epsilon \sim \mathcal{N}(0,1)$ is the prior Gaussian distribution that allows for backpropagation through the stochastic sampling process. The sampled latent variables vector $\mathbf{z}$ is then passed to the decoder.

Decoder. The latent space is up-sampled by two linear layers to sizes of 1024 and 6400, respectively, before being reshaped to dimensions of $N \times 32 \times 200$, where N is the batch size. This reshaped tensor goes into a transpose convolutional layer with a kernel size of 7, stride of 1, and padding of 3 to a shape of $N \times 128 \times 200$. The output from the convolutional decoder layer enters two transformer decoder blocks in series, each of which has two multi-headed self-attention layers in series, layer normalization, and feedforward layers as shown in Figure S3. A convolutional layer at the output of the transformer decoder reduces the size of the tensor to the size of the output trajectory, which is equal to the size of the input trajectory.

Loss function. The loss function consists of a total of 11 terms; all summed together with their respective weights determined based on the first epoch losses as defined below.

$$\mathcal{L} = \sum_{j=1}^{10} w_j \times \mathcal{L}_j, \tag{S6}$$

where, w_j and $\mathcal{L}_j$ are the weights and loss components j, respectively. Each loss function component is defined in the following set of equations. The mean squared error (MSE) loss between the input and generated trajectories is defined first as the L2 norm:

$$\mathcal{L}_1 = \frac{1}{N}||\mathbf{r} - \hat{\mathbf{r}}||_2^2, \tag{S7}$$

where $N = 1000$ is the batch size.

The KL-divergence loss, as defined below, ensures that the posterior distribution of latent variables adheres to the prior Gaussian distribution:

$$\mathcal{L}_2 = D_{\mathrm{KL}}(q(\mathbf{z}|\mathbf{r})||p(\mathbf{z})) = \int q(\mathbf{z}|\mathbf{r}) \log \frac{q(\mathbf{z}|\mathbf{r})}{p(\mathbf{z})}\, d\mathbf{z}, \tag{S8}$$

where $q(\mathbf{z}|\mathbf{r})$ is the approximate posterior distribution of latent variables $\mathbf{z}$ given the input trajectory $\mathbf{r}$, and $p(\mathbf{z})$ is a standard normal distribution $\mathcal{N}(0,1)$.

The next four equations describe the loss components of the moments of the distributions of displacements of the trajectories. In each case, a mean squared error was taken between the moments of the input trajectory and the moments of the reconstructed trajectory.

$$\mathcal{L}_3 = \frac{1}{N}||\langle\delta\mathbf{r}\rangle - \langle\delta\hat{\mathbf{r}}\rangle||_2^2, \tag{S9}$$

where $\langle\delta\mathbf{r}\rangle$ is the mean, μ_{δ_r}, of the distribution of displacements of the input trajectory, and $\langle\delta\hat{\mathbf{r}}\rangle$ is the mean, $\mu_{\delta\hat{r}}$, of the distribution of displacements of the reconstructed trajectory.

$$\mathcal{L}_4 = \frac{1}{N}||\langle(\delta\mathbf{r} - \mu_{\delta r})^2\rangle - \langle(\delta\hat{\mathbf{r}} - \mu_{\delta\hat{r}})^2\rangle||_2^2, \tag{S10}$$

$$\mathcal{L}_5 = \frac{1}{N}\left|\left|\frac{\langle(\delta\mathbf{r} - \mu_{\delta r})^3\rangle}{\langle\sigma_{\delta r}\rangle^3} - \frac{\langle(\delta\hat{\mathbf{r}} - \mu_{\delta\hat{r}})^3\rangle}{\langle\sigma_{\delta\hat{r}}\rangle^3}\right|\right|_2^2, \tag{S11}$$

$$\mathcal{L}_6 = \frac{1}{N}\left|\left|\frac{\langle(\delta\mathbf{r} - \mu_{\delta r})^4\rangle}{\langle\sigma_{\delta r}^2\rangle^2} - \frac{\langle(\delta\hat{\mathbf{r}} - \mu_{\delta\hat{r}})^4\rangle}{\langle\sigma_{\delta\hat{r}}^2\rangle^2}\right|\right|_2^2, \tag{S12}$$

where $\sigma_{\delta r}$ and $\sigma_{\delta\hat{r}}$ are the standard deviation of $\delta\mathbf{r}$ and $\delta\hat{\mathbf{r}}$ distributions, respectively, and $\langle\cdot\rangle$ denotes an average over the trajectory displacements.

The loss comparing the medians of the distributions of displacements for the input and reconstructed trajectories is defined as:

$$\mathcal{L}_7 = \frac{1}{N}||(\tilde{\delta \mathbf{r}}) - (\tilde{\delta \hat{\mathbf{r}}})||_2^2, \tag{S13}$$

where $(\tilde{\delta \mathbf{r}})$ represents the median of the displacement distribution of the input trajectory, and $(\tilde{\delta \hat{\mathbf{r}}})$ represents the median of the displacement distribution of the reconstructed trajectory.

The velocity autocorrelation loss component is defined for $\tau = 1$ to $\tau = 50$ (the first 50 time delays of $\mathbf{C_v}$), with each time delay weighted by $1/\tau$:

$$\mathcal{L}_8 = \frac{1}{N}\sum_{\tau=1}^{50}\frac{1}{\tau}||C_{\mathbf{v}}(\tau) - C_{\hat{\mathbf{v}}}(\tau)||_2^2, \tag{S14}$$

where $C_{\mathbf{v}}(\tau) = \frac{\langle \mathbf{v}(t)\cdot\mathbf{v}(t+\tau)\rangle}{\langle \mathbf{v}^2(t)\rangle}$ refers to the velocity autocorrelation of the input trajectories, with $C_{\hat{\mathbf{v}}}(\tau) = \frac{\langle \hat{\mathbf{v}}(t)\cdot\hat{\mathbf{v}}(t+\tau)\rangle}{\langle \hat{\mathbf{v}}^2(t)\rangle}$ referring to the velocity autocorrelation of the reconstructed trajectories. The weighting factor of $1/\tau$ emphasizes the importance of short time lags (τ), which are more relevant for understanding the viscoelasticity of the interaction energy landscape investigated in this study. Longer time lags contribute less to the overall loss, as they are less informative for our study and are statistically less reliable due to the finite length of the trajectories. This weighting is particularly appropriate because the velocity autocorrelation function is most meaningful when $\tau \ll T$, where T is the total length of the trajectory.

The point-wise ensemble-averaged velocity autocorrelation is another important statistical measure that measures the correlations of an ensemble of particle trajectories at each time delay τ. For example, particle trajectories from LPTEM usually have a negative value for the velocity autocorrelation at short-time delays τ, which can be seen in the point-wise ensemble-averaged velocity autocorrelation of trajectories at short-time delays. These trajectories also have zero correlations at longer time delays, which ensures the stochasticity of the trajectories, *i.e.*, there are no predictable correlations within particle trajectories after the initial correlations at shorter time delays. To define the point-wise ensemble-averaged velocity autocorrelation, for each batch of trajectories in model training, we calculated the mean squared error between the velocity autocorrelation of the input batch, ensemble-averaged over the input batch, and the velocity autocorrelation of the generated batch, ensemble-averaged over the generated batch. This was then averaged across all time delays to obtain a singular value for the error as defined below:

$$\mathcal{L}_9 = \overline{(\langle \mathbf{C_v}\rangle - \langle \mathbf{C_{\hat{v}}}\rangle)^2}. \tag{S15}$$

where $\langle\cdot\rangle$ denotes average over a batch of N trajectories in the training dataset, and $\overline{(\cdot)}$ denotes the average over time delay windows of size $\tau = 1$ to $\tau = T - 2$ with $T = 200$ in this case.

The correlation between the x and y components of the 2-D trajectories is accounted for by a loss term that measures the deviation in the correlation coefficient between the input and reconstructed trajectories. This term ensures that the model captures any anisotropy or coupling between orthogonal motion components, which is particularly important for 2-D trajectories.

$$\mathcal{L}_{10} = \frac{1}{N}||\rho_{\delta\mathbf{x},\delta\mathbf{y}} - \rho_{\delta\hat{\mathbf{x}},\delta\hat{\mathbf{y}}}||_2^2, \tag{S16}$$

where $\rho_{\delta\mathbf{x},\delta\mathbf{y}}$ is the correlation coefficient between the x and y displacements of the input trajectories, and $\rho_{\delta\hat{\mathbf{x}},\delta\hat{\mathbf{y}}}$ is the corresponding correlation coefficient for the reconstructed trajectories. The correlation coefficient for each trajectory is computed as:

$$\rho_{\delta\mathbf{x},\delta\mathbf{y}} = \frac{\mathrm{Cov}(\delta\mathbf{x},\delta\mathbf{y})}{\sqrt{\sigma^2_{\delta\mathbf{x}}\cdot\sigma^2_{\delta\mathbf{y}}}}, \tag{S17}$$

where $\mathrm{Cov}(\delta\mathbf{x},\delta\mathbf{y}) = \frac{1}{N}\sum_{i=1}^{N}(\delta x_i - \langle\delta x\rangle)(\delta y_i - \langle\delta y\rangle)$ represents the covariance between the x and y displacements, and $\sigma^2_{\delta\mathbf{x}}$ and $\sigma^2_{\delta\mathbf{y}}$ represent the variances of the x and y displacements, respectively, and $\langle\cdot\rangle$ denotes the average over the trajectory displacements.

By including this term, the model is encouraged to reproduce the same level of anisotropy or coupling between x and y components as observed in the input trajectories. This is critical for accurately modeling complex systems where the motion in orthogonal directions may not be independent or isotropic.

The positional autocorrelation loss component compares the spatial correlation between particle positions at different time lags for the input and reconstructed trajectories. The positional autocorrelation

function is defined as $C_{\mathbf{r}}(\tau) = \frac{\langle \mathbf{r}(t)\cdot\mathbf{r}(t+\tau)\rangle}{\langle \mathbf{r}^2(t)\rangle}$, where $\mathbf{r}(t)$ and $\mathbf{r}(t+\tau)$ represent the position vectors at times t and $t+\tau$, respectively. The loss term is defined as:

$$\mathcal{L}_{11} = \frac{1}{N}\sum_{\tau=1}^{T-1} ||\mathbf{C}_{\mathbf{r}}(\tau) - \mathbf{C}_{\hat{\mathbf{r}}}(\tau)||_2^2, \tag{S18}$$

where $\mathbf{C}_{\mathbf{r}}(\tau)$ represents the positional autocorrelation of the input trajectories, and $\mathbf{C}_{\hat{\mathbf{r}}}(\tau)$ represents the positional autocorrelation of the reconstructed trajectories. The inclusion of this term was motivated by the distinct behaviors observed in our experimental trajectories, where particles often transition abruptly between positions and remain localized in the new positions for extended periods. These dynamics introduce long-term spatial correlations that are not fully captured by displacement-based metrics.

The weights chosen for each loss term component based on the magnitude of the first epoch losses were $w_1 = 0.001, w_2 = 0.05, w_3 = 50,000, w_4 = 500, w_5 = 10, w_6 = 0.06, w_7 = 1, w_8 = 1000, w_9 = 100, w_{10} = 10, w_{11} = 1$. The very low weight assigned to w_1 (corresponding to the MSE-based reconstruction loss) emphasizes that the contribution of the MSE loss term is small compared to the physics-informed loss terms. This choice of weight reflects our focus on reproducing the statistical distribution of the trajectories rather than achieving an exact point-wise reconstruction.

3.2 Model training

All the parameters of the LEONARDO model architecture were trained by backpropagating the derivative of the loss function with respect to the model parameters using the ADAM optimizer with a learning rate of 3×10^{-4}.

3.3 Model validation

Separate sets of experimental trajectories were segmented and sub-sampled for validation and testing using the methodology described in Section 2 of the SI. This resulted in $3,202$ trajectories for validation and $5,934$ trajectories for testing. Validation was performed to tune hyperparameters and reconstruct losses at each epoch during model training to compare against the training losses as described in Methods Section 4.3. These trajectories were not used to update the model parameters during backpropagation. The test set was used to report the final model performance. Figure S1 shows the validation losses per epoch averaged over the 3202 trajectories for each loss component of LEONARDO and their comparison with respect to the training loss at each epoch.

4 Supporting Tables

	LEONARDO-Generated *vs.*							LEONARDO-Reconstructed *vs.*
**Metric**	**LPTEM**	**Brownian**	**FBM**	**CTRW**	**Lévy Walk**	**SBM**	**ATTM**	**LPTEM**
Mean (10^{-7})	5.13	0.25	0.24	0.80	0.20	0.30	1.09	0.27
Variance (10^{-6})	8.30	41.20	26.10	31.80	63.90	40.90	38.40	2.59
Skewness (10^{-2})	1.06	0.03	0.02	0.04	0.07	0.03	0.15	0.05
Kurtosis	0.21	20.27	20.28	4432.95	24.61	5.96	280.20	0.19
Median (10^{-8})	19.58	6.01	8.49	6.31	9.56	5.28	11.00	21.93
Velocity Autocorr (10^{-6})	1.11	21.70	0.77	22.00	713.98	21.80	21.60	0.01
Batch Velocity Autocorr (10^{-6})	2.05	15.00	0.00	24.10	4126.94	19.80	20.70	0.92
XY Correlation (10^{-5})	2.61	23.41	25.50	47.51	30.81	21.83	7.82	0.01
Position Autocorr (10^{-3})	1.04	7.90	4.50	0.08	28.16	4.57	1.34	0.70
Total Weighted Squared Error	**2.33**	48.03	2.97	312.43	649.39	46.86	63.29	**0.14**
Total Unweighted Squared Error	**0.22**	20.28	20.28	4432.95	24.65	5.96	280.20	**0.19**

Table S1: Statistical comparison of LEONARDO-generated and LEONARDO-reconstructed trajectories against experimental LPTEM trajectories and reference diffusion classes. The first seven columns (LEONARDO-generated) report the squared differences between the average statistical properties of LEONARDO-generated trajectories (sampled randomly from the latent space) and those of experimental LPTEM trajectories, as well as independent batches of Brownian motion, FBM, CTRW, Lévy Walk, SBM, and ATTM. The last column reports the squared differences between the same LPTEM trajectories as the first column, and LEONARDO-reconstructed trajectories, obtained by inputting the LPTEM trajectories into the trained model. This column highlights the high fidelity of LEONARDO's reconstructions, which yield significantly lower errors for most statistical properties compared to the reference errors observed for LEONARDO-generated trajectories against LPTEM and other diffusion classes. Each metric is computed over an ensemble of 3,202 trajectories. The last two rows summarize the total weighted (using the same weights as in the LEONARDO training loss; see Section 3.1 of the SI) and unweighted squared errors. Lower values indicate higher statistical similarity.

Latent Variable	z_1	z_2	z_3	z_4	z_5	z_6	z_7	z_8	z_9	z_{10}	z_{11}	z_{12}
σ^2	0.011	0.95	0.26	0.067	0.0088	0.11	0.96	0.12	0.96	0.065	0.97	0.94

Table S2: Variances of the latent variables from the encoder in LEONARDO, averaged over the test set. These variances indicate which latent variables are encoding meaningful statistical properties of the trajectories. Latent variables with low variances, *i.e.*, around 1-2 orders of magnitude lower than unity (*e.g.*, z_1, z_4, and z_5), encode key properties such as non-Gaussianity and velocity autocorrelation. Other latent variables with low variances encode additional properties not directly related to the statistical properties of interest in this work.

5 Supporting Figures

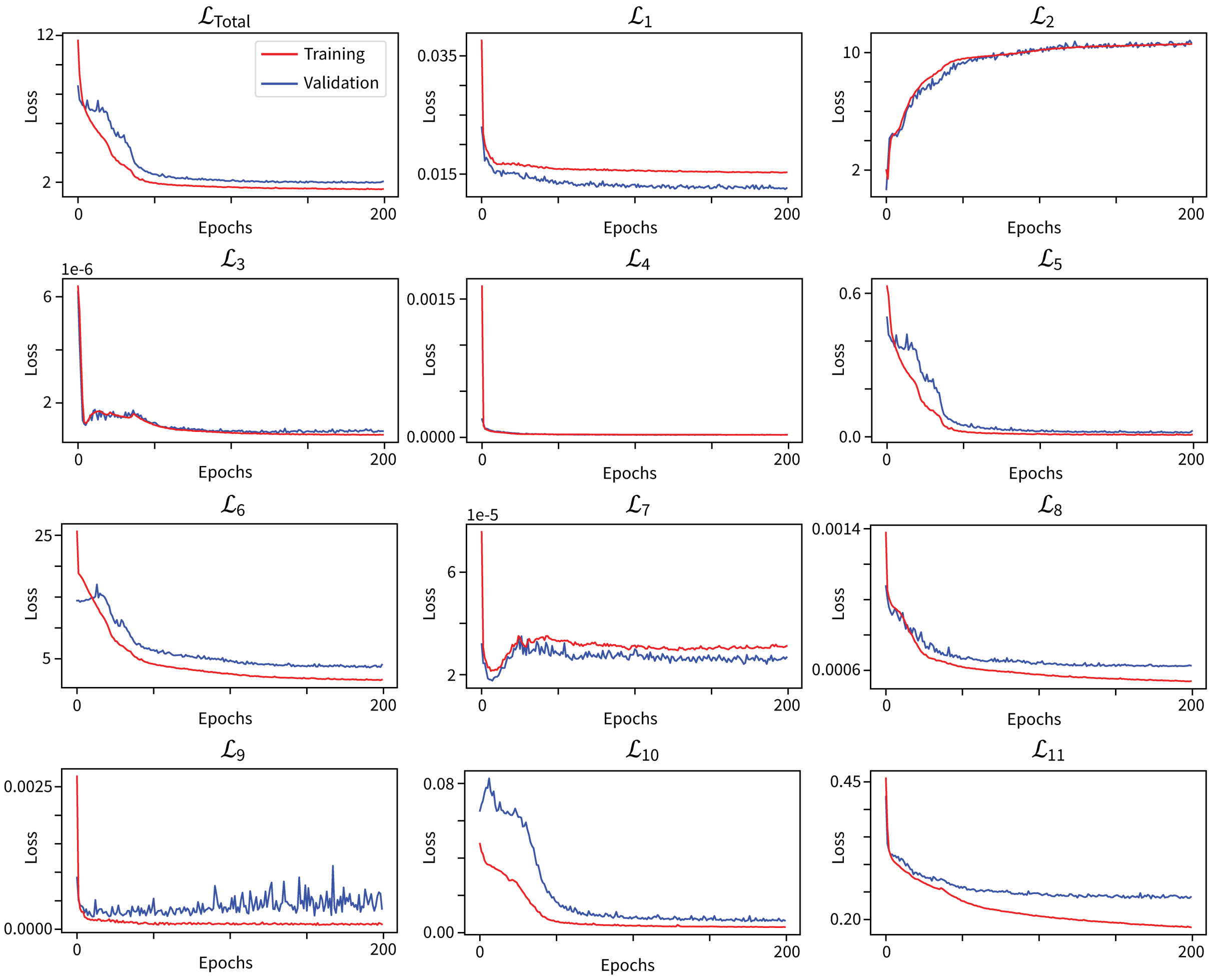

Fig. S1: LEONARDO training and validation losses for each loss component at each epoch

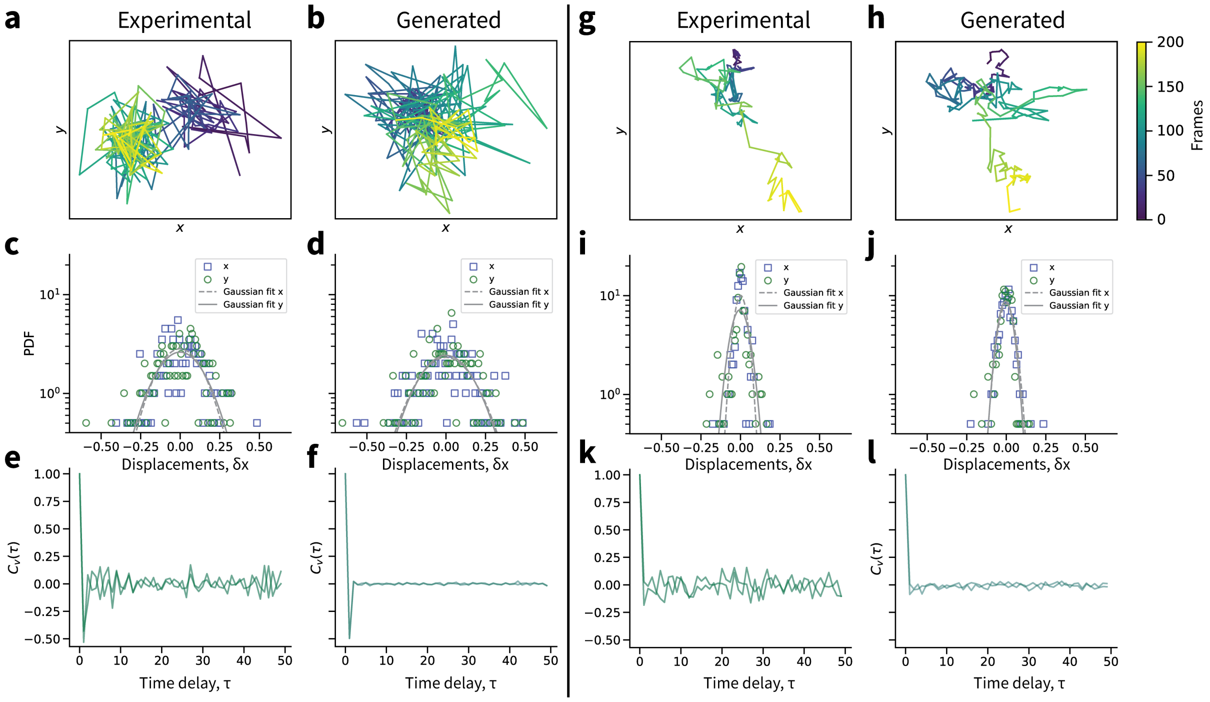

Fig. S2: **Comparison of experimental and synthetic trajectories generated using LEONARDO.** **a**, Example experimental trajectory from LPTEM test dataset with a high velocity autocorrelation at $\tau = 1$. **b**, Trajectory in (a) encoded and decoded by LEONARDO to generate a synthetic trajectory with similar statistical properties to (a). **c**, x and y-component distribution of displacements for trajectory in (a). **d**, x and y-component distribution of displacements for trajectory in (b). **e**, x and y-component velocity autocorrelation plots for trajectory in (a). **f**, x and y-component velocity autocorrelation plots for trajectory in (a). **g-l**, Same analysis as (a)-(f) for an example experimental trajectory from LPTEM test dataset with low velocity autocorrelation at $\tau = 1$.

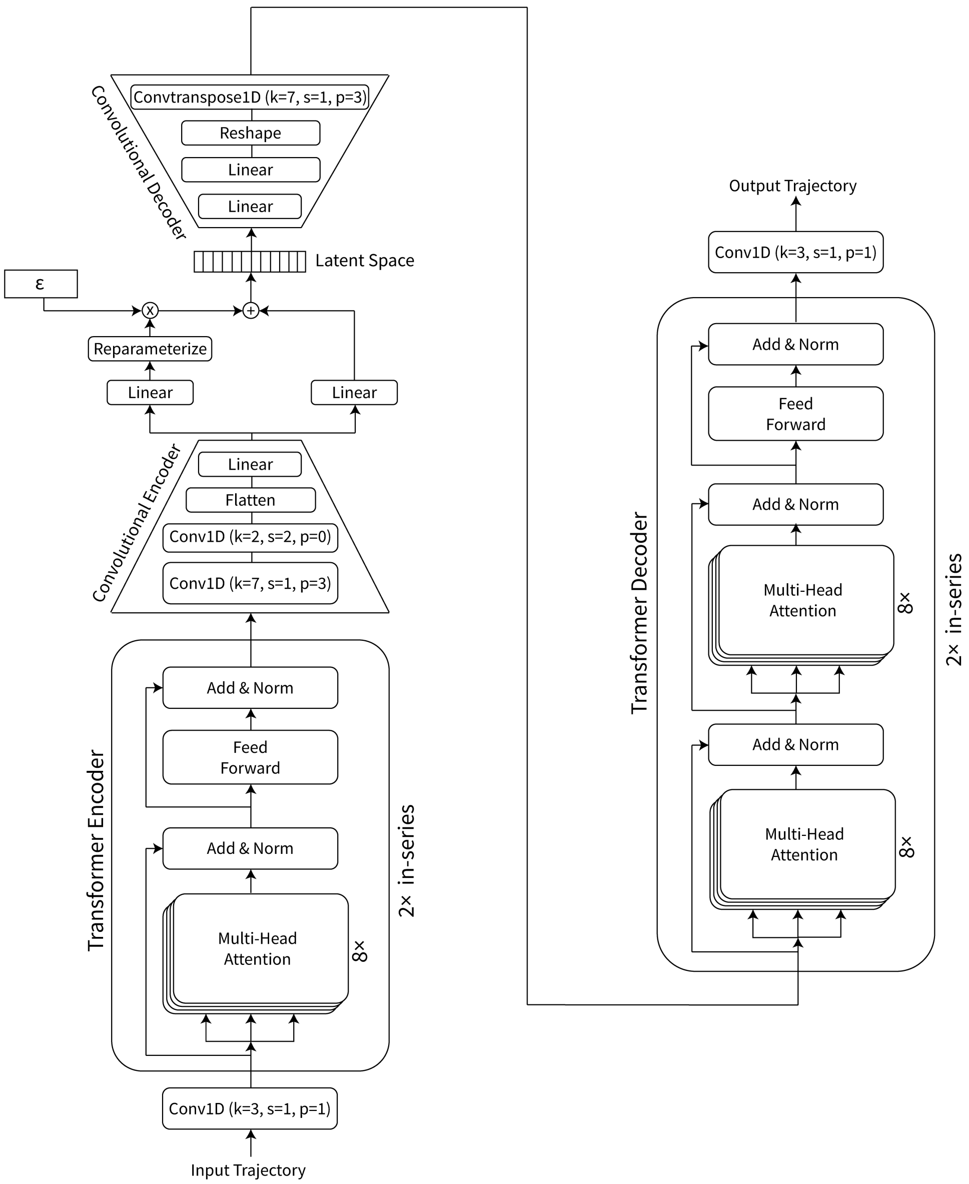

Fig. S3: **LEONARDO model architecture**

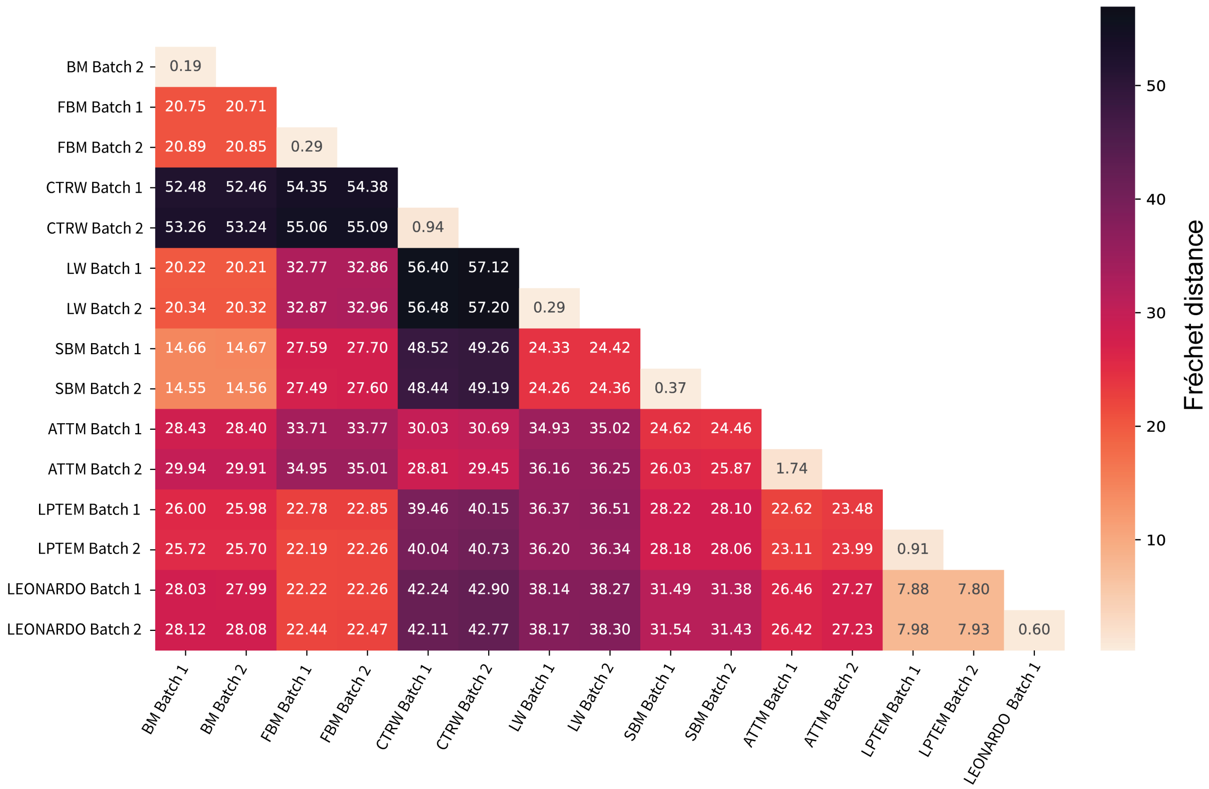

Fig. S4: **Fréchet Distance lower triangular matrix showing scores between pairs of diffusion class including intra-class scores.** The second-last layer output of MoNet2.0 is used to compute the FD scores between different diffusion classes and between batches of the same class (intra-class scores). The matrix shows that the FD scores between LPTEM and LEONARDO-generated trajectories are significantly lower than the scores between other diffusion classes, while intra-class scores, ranging from 0.19 to 1.74, provide a lower bound for contextual comparison.

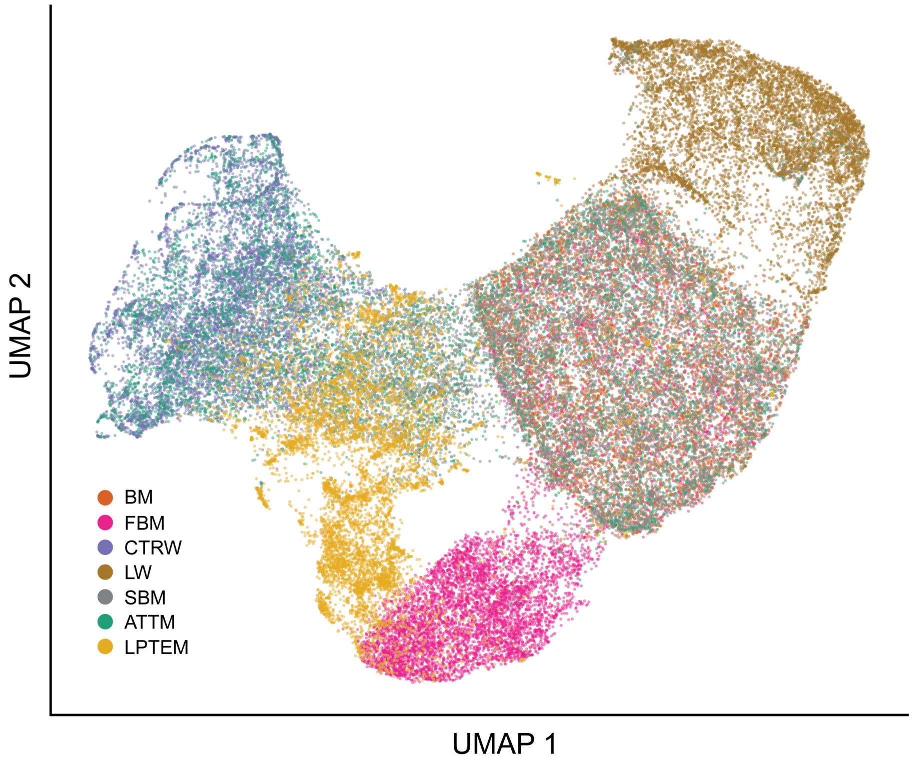

Fig. S5: **UMAP of LEONARDO latent space with AnDi simulated trajectories and experimental LPTEM trajectories encoded**

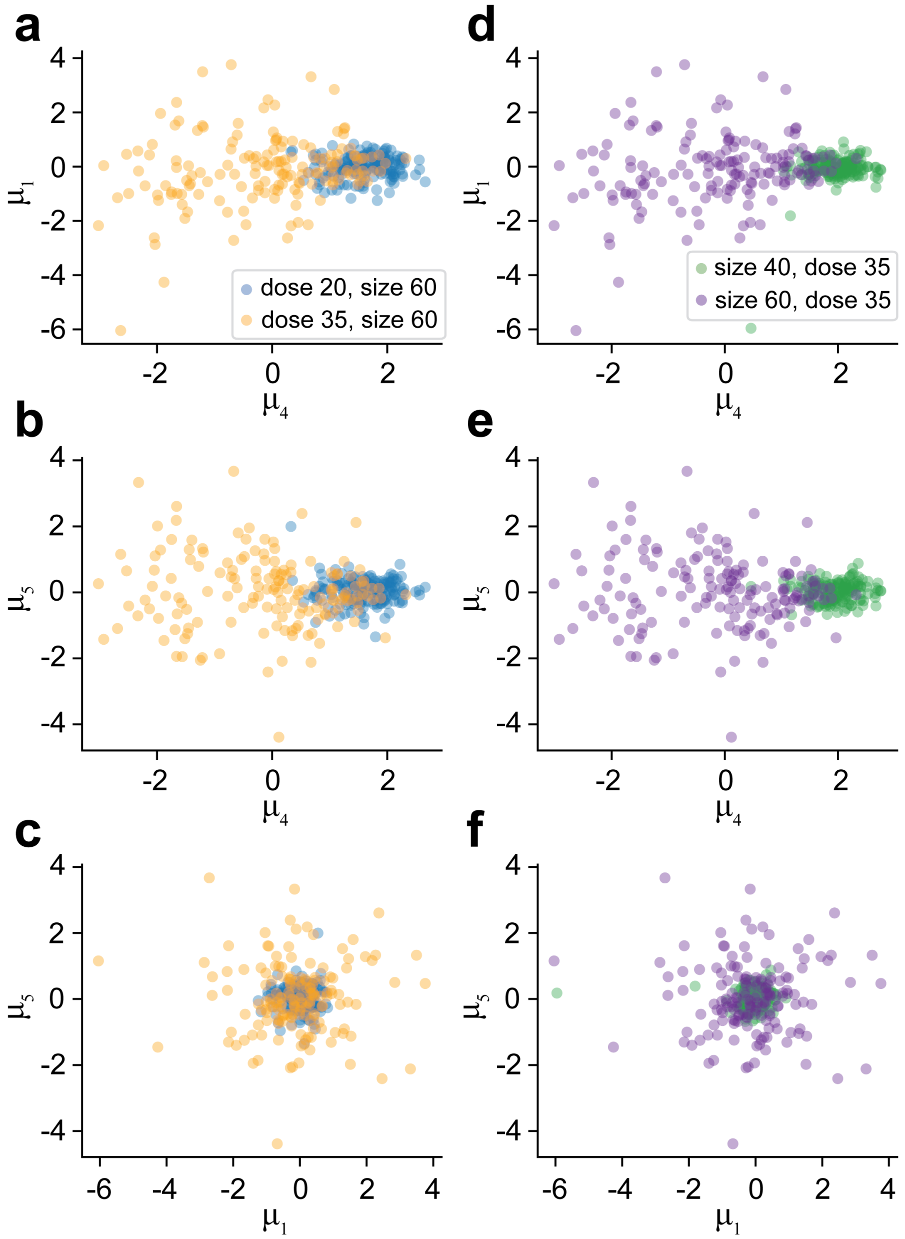

Fig. S6: **LEONARDO latent space scatter plots.** **a**, Scatter plot of the values of μ_4 versus μ_1 for experimental trajectories collected at electron beam dose rates of 20 and 35 $e^-/Å^2$·s for a fixed particle size of 60 nm. **b**, Scatter plot of the values of μ_4 versus μ_5 for the same experimental trajectories as in (a). **c**, Scatter plot of the values of μ_1 versus μ_5 for the same experimental trajectories as in (a) and (b). **d**, Scatter plot of the values of μ_4 versus μ_1 for experimental trajectories collected at particle sizes of 40 nm and 60 nm at a fixed electron beam dose rate of 35 $e^-/Å^2$·s. **e**, Scatter plot of the values of μ_4 versus μ_5 for the same experimental trajectories as in (d). **f**, Scatter plot of the values of μ_1 versus μ_5 for the same experimental trajectories as in (d) and (e).

References

[1] Nikoobakht, B. & El-Sayed, M. A. Preparation and growth mechanism of gold nanorods (nrs) using seed-mediated growth method. *Chemistry of materials* **15**, 1957–1962 (2003).

[2] Ye, X., Chen, J., Diroll, B. T. & Murray, C. B. Tunable plasmonic coupling in self-assembled binary nanocrystal superlattices studied by correlated optical microspectrophotometry and electron microscopy. *Nano letters* **13**, 1291–1297 (2013).

[3] Vaswani, A. *et al.* Attention is all you need. *Advances in neural information processing systems* **30** (2017).

[4] Kingma, D. P. & Welling, M. Auto-encoding variational bayes. *arXiv preprint arXiv:1312.6114* (2013).
